# Clinical and prognostic implications of an immune‐related risk model based on *TP53* status in lung adenocarcinoma

**DOI:** 10.1111/jcmm.17097

**Published:** 2021-12-08

**Authors:** Xuming Song, Qiang Chen, Jifan Wang, Qixing Mao, Wenjie Xia, Lin Xu, Feng Jiang, Gaochao Dong

**Affiliations:** ^1^ The Affiliated Cancer Hospital of Nanjing Medical University Nanjing China; ^2^ Department of Thoracic Surgery Jiangsu Cancer Hospital Jiangsu Institute of Cancer Research Nanjing China; ^3^ Jiangsu Key Laboratory of Molecular and Translational Cancer Research Cancer Institute of Jiangsu Province Nanjing China; ^4^ The Fourth Clinical College of Nanjing Medical University Nanjing China; ^5^ XuZhou Central Hospital Xuzhou China

**Keywords:** immune profile, immune prognostic model, lung adenocarcinoma, TP53 mutation

## Abstract

*TP53* mutation is the most widespread mutation in lung adenocarcinoma (LUAD). Meanwhile, p53 (encoded by *TP53*) has recently been implicated in immune responses. However, it is still unknown whether *TP53* mutation remodels the tumour microenvironment to influence tumour progression and prognosis in LUAD. In this study, we developed a 6‐gene immune‐related risk model (IRM) to predict the survival of patients with LUAD in The Cancer Genome Atlas (TCGA) cohort based on *TP53* status, and the predictive ability was confirmed in 2 independent cohorts. *TP53* mutation led to a decreased immune response in LUAD. Further analysis revealed that patients in the high‐index group had observably lower relative infiltration of memory B cells and regulatory T cells and significantly higher relative infiltration of neutrophils and resting memory CD4^+^ T cells. Additionally, the IRM index positively correlated with the expression of critical immune checkpoint genes, including *PDCD1* (encoding PD‐1) and *CD274* (encoding PD‐L1), which was validated in the Nanjing cohort. Furthermore, as an independent prognostic factor, the IRM index was used to establish a nomogram for clinical application. In conclusion, this IRM may serve as a powerful prognostic tool to further optimize LUAD immunotherapy.

## 1 INTRODUCTION

Non‐small cell lung cancer (NSCLC) accounts for 85% of all lung cancers, and lung adenocarcinoma (LUAD) is the most frequent NSCLC subtype.^1^ Immunotherapy has been integrated into the first‐ and second‐line treatment strategies for NSCLC, reviving enthusiasm in explaining the prognostic and pathophysiological role of the tumour microenvironment (TME).^2^, ^3^, ^4^, ^5^ However, it is not known whether the immune signature of lung cancer could act as a biomarker to reliably estimate disease prognosis and patient survival.

*TP53* is a tumour suppressor gene, and it is one of the 5 most conspicuous mutations in LUAD, though there has not yet been approval for any related molecular inhibitors by the Food and Drug Administration. *TP53* encodes p53, a master regulatory transcription factor that controls multiple core programs in cells, including cell cycle arrest, apoptosis, senescence, fertility and metabolism.^6^, ^7^, ^8^, ^9^, ^10^ Recently, p53 has been implicated in immune responses.^11^ *TP53* mutation remarkably affects the expression of immune checkpoints and activated T‐cell immune response and may thus serve as a potential predictive factor for guiding immunotherapy.^11^ However, the relationship between *TP53* mutation and the regulation of immune signalling and responses is still unknown. We hypothesized that *TP53* mutation may remodel the TME to influence tumour progression and prognosis in LUAD.

Recently, computational methods based on transcriptome data were proposed to characterize the immune landscape in the sequenced tumour tissue.^12^ In this study, we systemically screened the expression profiles of RNA sequencing data from public databases and developed a 6‐gene immune‐related risk model (IRM) based on *TP53* mutation to predict the survival of patients with LUAD. We further validated the 6‐gene IRM index in the meta‐Gene Expression Omnibus (GEO) and Nanjing cohorts. Through multiple verification methods, we determined the IRM index to be an independent prognostic biomarker that could accurately predict the 5‐ and 7‐year overall survival (OS) of patients with LUAD. The potential mechanism, immune infiltration and expression of immune‐related checkpoints of the IRM index were also explored. We believe that this robust prognostic IRM index will improve risk stratification, provide more exact judgment for individualized clinical management, and serve as a potential immunotherapy biomarker for patients with LUAD.

## 2 MATERIALS AND METHODS

### 2.1 RNA sequencing data and immune‐related genes

The nonsynonymous mutation status of 512 patients with LUAD (Mutect2 pipeline), gene expression counts matrix (HTSeq‐Counts) and corresponding clinical metadata from The Cancer Genome Atlas (TCGA) website (https://gdc.cancer.gov/) which downloaded from the ‘TCGAbiolinks’ R package (version 2.14.1). Of these patients, 499 with mRNA relative expression and Whole exon sequencing data were included in the following analysis. We identified the *TP53* mutation status of these patients via the ‘maftools’ R package (version 2.2.9), including 239 TP53^MUT^ patients and 260 TP53^WT^ patients.

### 2.2 Differential expression analysis

Differential expression analysis was performed using the ‘DESeq2’ R package (version 1.26.0) with the standard comparison mode between different *TP53* mutation status. To filter differentially expressed genes (DEGs) between TP53^mut^ and TP53^wt^ patients, log2|fold change| >1 and adj. *p*.value < 0.05 were applied as the cut‐off point.

### 2.3 Construction of the IRM index

Immune‐related differentially expressed genes (IRDEGs) were collected from the ImmPort database (http://www.immport.org), which includes annotated genes related to immune activity processes. After identifying IRDEGs, a LASSO algorithm was built using the ‘glmnet’ R package (version 3.0) to select candidate genes. We conducted Kaplan‐Meier survival curves using the R package ‘survival’ (version 3.5). Finally, the ‘timeROC’ R package (version 0.4) was used to conduct a time‐dependent receiver operating characteristic (ROC) curve analysis.

### 2.4 Microarray data

The gene expression matrixes from GSE29013, GSE30219, and GSE31908 based on the GPL570 platform, including 131 patients with LUAD, were downloaded from the GEO database (https://www.ncbi.nlm.nih.gov/gds). The gene expression data for the 3 matrixes were subjected to log_2_ transformation. We used ‘combat’ function from ‘sva’ R package (version 3.36) to remove the batch effects.

### 2.5 Patients in the Nanjing cohort and sample collection

To further evaluate the clinical effectiveness of the IRM index, we enrolled a cohort including 92 patients who were diagnosed with LUAD at the Jiangsu Cancer Hospital (Nanjing, China) and underwent radical surgery without neoadjuvant chemotherapy from 2012 to 2018. The patients’ characteristics are presented in Table S1. Tissue samples of patients in the Nanjing cohort were collected from surgical formalin‐fixed, paraffin‐embedded (FFPE) specimens.

### 2.6 RNA extraction and qRT‐PCR

We extracted total RNA via the RNeasy FFPE Kit (Qiagen, #73504) from 4‐μm‐thick FFPE specimens. We used PrimeScript RT Master Mix (Takara, #RR035A) reverse transcription of the complementary DNA (cDNA). We performed the qRT‐PCR assays using the ViiA 7 Dx RT‐PCR System (Applied Biosystems) with AceQ qPCR SYBR Green Master Mix (Vazyme, #Q111‐02). The internal control of target genes GAPDH using the 2^‐ΔΔCT method. The primer sequences are provided in Table S2. We used ‘combat’ function from ‘sva’ R package (version 3.36) to remove the batch effects.

### 2.7 Function enrichment analysis

Metascape (https://metascape.org/) ^13^ is an online pathway enrichment analysis visualization tool. We performed Gene Ontology (GO) and The Kyoto Encyclopedia of Genes and Genomes (KEGG) pathway analyses for DEGs and IRDEGs using Metascape. Select the most enriched set of genes in the cluster as one of the representative clusters.

### 2.8 Gene set enrichment analysis and principal components analysis

To determine the potential immune‐related pathways and genes between TP53^mut^ and TP53^wt^ patients with LUAD in the TCGA cohort, gene set enrichment analysis (GSEA) (version 4.0.0) was performed. An annotated gene set file (msigdb.v7.0.entrez.gmt) was selected for use as the reference gene set. We carried out a principal components analysis (PCA) via R package ‘pca3d’ (version 0.10.1).

### 2.9 CIBERSORTx analysis

The CIBERSORTx website (https://cibersort.stanford.edu/index.php) provides an algorithm to quantify the relative infiltration of several immune‐infiltrating cells in the TME,^12^ including T‐cell subtypes, naive and memory B cells, myeloid cell subsets, natural killer (NK) cells and plasma cells. The data of immune‐infiltrating cell infiltration levels in patients with LUAD were extracted from CIBERSORTx to investigate the correlation with the IRM index.

### 2.10 Immunohistochemistry

Formalin‐fixed paraffin‐embedded lung adenocarcinoma specimens from the Nanjing cohort were serially sectioned at 4‐μm‐thick spacings and then mounted on glass slides. In brief, we used ethylenediaminetetraacetic acid (EDTA) antigen retrieval buffer boiling to deparaffinize the sections and then incubated them with antibodies (anti‐PD‐1 antibody, abcam, #ab237728; anti‐PD‐L1 antibody, abcam, #ab205921) overnight at 4°C. The sections were subsequently analysed using the HRP‐Polymer anti‐Rabbit IHC Kit (MaxVision, #KIT‐5005). Immunohistochemistry (IHC) staining results were assessed and scored independently by 2 senior pathologists. The expression of PD‐1 and PD‐L1 in the tumour was determined as the proportion of positively coloured cells compared to the total number of cells counted.

### 2.11 Nomogram construction and validation

Univariate and multivariate Cox analyses were used to evaluate the IRM index as an independent prognostic factor. Then, a concise nomogram to predict the OS of LUAD was established using the ‘rms’ R package (version 2.10; https://cran.r‐project.org/web/packages/rsm/index.html), including 4 factors. We conducted 5‐ and 7‐year OS alignments to determine the prognostic ability of the nomogram model.

### 2.12 Statistical analysis

Statistical analyses were performed using R (version 3.6.1). The Student's *t*‐test was used to determine differences for 2‐group comparisons.

## 3 RESULTS

### 3.1 Immune landscape based on *TP53* mutations in LUAD

**FIGURE 1:**
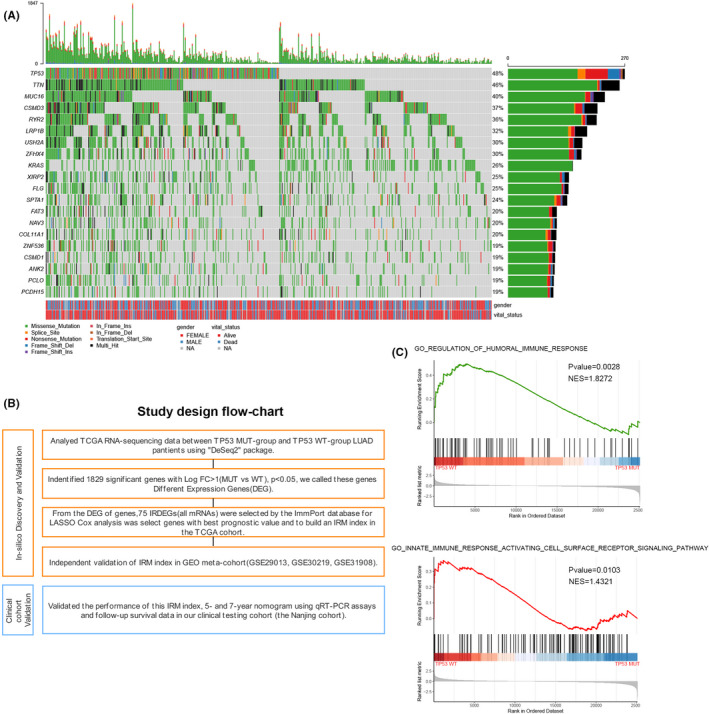
Study design and in silico discovery of TP53‐associated genes. (A) Genomic landscape of lung adenocarcinoma and the mutational signature in the TCGA dataset. (B)The study design. (C) Significant enrichment of the immune‐related phenotype in TP53MUT LUAD patients compared with that in TP53WT LUAD patients by GSEA

In LUAD, *TP53* mutation is the most widespread somatic nonsynonymous mutation (Figure 1A). To evaluate the correlation between immune infiltration and *TP53* mutation in patients with LUAD, a flowchart was constructed to reveal the analysing process (Figure 1B). LUAD patients in the TCGA cohort were separated into 2 groups: TP53^WT^ (260 patients) and TP53^MUT^ (239 patients). Subsequently, we performed DEG analysis based on *TP53* mutations, which revealed that 1829 genes were statistically differentially expressed between the TP53^MUT^ group and the TP53^WT^ group, including 1230 downregulated genes and 599 upregulated genes (Figure S1A, Table S3).

Metascape was used to annotate potential pathways, and it showed that the DEGs mentioned above were mainly involved in pathways related to immunology (Figure S1B). Next, to further prove the correlation between DEGs and tumour immunology, GSEA was used to show that TP53^wt^ patients with LUAD were enriched in 324 biological processes, including 2 immune‐related functional pathways: GO_REGULATION_OF_HUMORAL_IMMUNE_RESPONSE and GO_INNATE_IMMUNE_ RESPONSE_ACTIVATING_CELL_SURFACE_RECEPTOR_SIGNALING_PATHWAY (Figure 1C), which further confirmed the potential correlation between immunology and *TP53* status.

### 3.2 IRM index construction and evaluation of its prognostic ability

**FIGURE 2:**
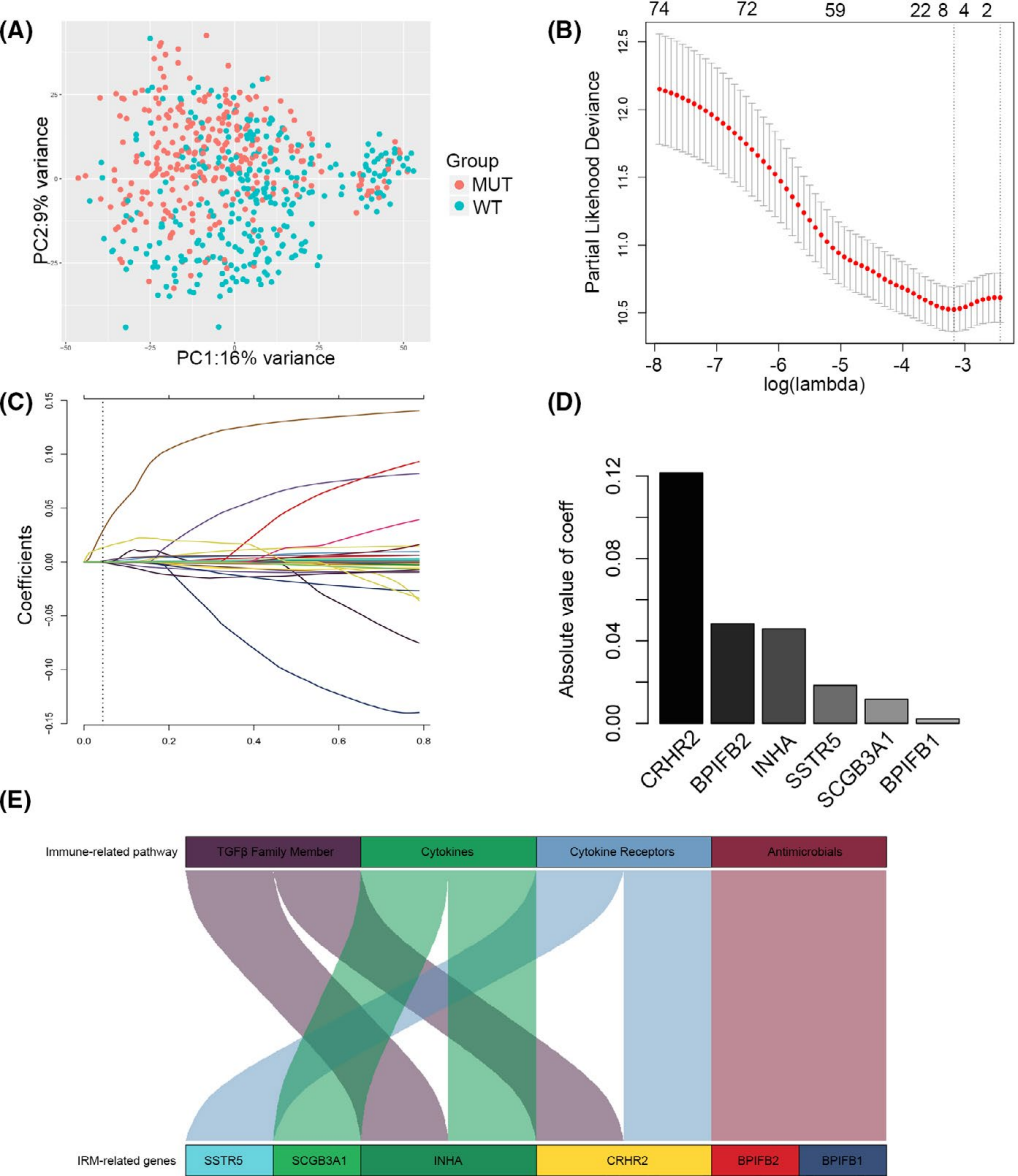
Identification of Immune‐Related Models Index (IRM index). (A) Principal components analysis performed on lung adenocarcinoma patients based on significant differences immune‐related RNA expressed between TP53MUT and TP53WT patients in the TCGA dataset. (B) Tuning parameter (lambda) screening in the LASSO regression model. (C) The LASSO coefficient profiles of the common genes. (D) Absolute value of coefficient for each of the six selected genes. (E) The immune‐related pathways enriched by IRM‐related six genes

To determine the relationship between *TP53* mutation status and immune‐related functional pathways, 75 IRDEGs were selected for further analysis from the 1829 DEGs based on the Immport database (Table S4). PCA indicated that these 75 IRDEGs could distinguish between the TP53^WT^ and TP53^MUT^ groups (Figure 2A). Next, we attempted to evaluate the prognostic performance of the 75 IRDEGs. We found six genes, including CRHR2, BPIFB2, INHA, SSTR5, SCGB3A1, and BPIFB1, with non‐zero regression concomitant coefficients and constructed a maximum prognostic value via LASSO and Cox analysis. (Figure 2B, C). Ultimately, a 6‐gene IRM was built, and the risk index of each patient was calculated using the following formula for further analysis: IRM index = (−0.121586693 × *CRHR2* expression) + (−0.048377824 × *BPIFB2* expression) + (0.045865315 × *INHA* expression) + (−0.018472910 × *SSTR5* expression) + (−0.011651852 × *SCGB3A1* expression) + (−0.002185301 × *BPIFB1* expression) (Figure 2D). The optimal cut‐point was −0.9595243. We annotated the immune activation pathway enriched by the 6 IRM‐related genes, which included transforming growth factor (TGF) family members, cytokines, cytokine receptors and antimicrobials (Figure 2E). Cancer cells and several immune‐suppressive cells in the TME recruit multiple cytokines, such as TGF‐β which facilitates tumour progression and mediates T‐cell dysfunction.^14^ This suggests that our IRM may be related to the immunosuppressive state of LUAD, which we will study next.

**FIGURE 3:**
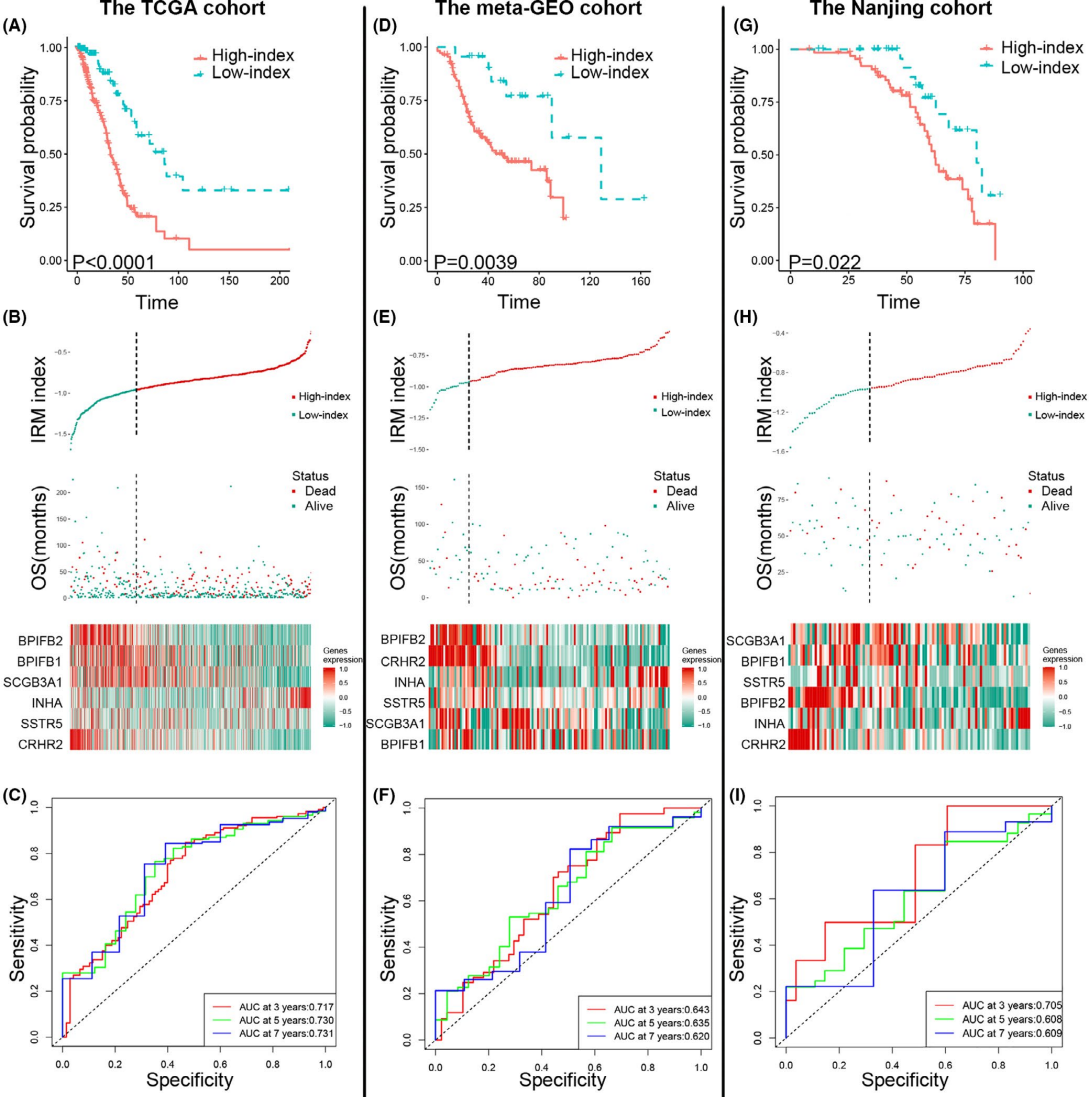
Prognostic analysis of the Immune‐Related Models Index (IRM index). Kaplan‐Meier survival, risk score, and time‐dependent ROC curves of TCGA cohort (A–C), meta‐GEO cohort (D–F), and the Nanjing cohort (G–I). (A, D and G) OS was significantly higher in the low‐index group than in the high‐index group. (B, E, and H) The relationship between the risk score (upper), the OS (middle), and the expression of six prognostic immune genes(bottom) is shown. (C, F and I) Time‐dependent ROC curve analysis of the IRM index

We separated patients with LUAD into the high‐ or low‐index groups across all 3 cohorts using the cut‐point in the TCGA cohort. The Kaplan‐Meier analysis illustrated that a high index was related to worse prognosis (Figure 3A). The IRM index distribution, OS, and 6‐gene expression heatmap are shown in Figure 3B. The time‐dependent ROC curve analysis of the IRM index in the TCGA cohort revealed the robustness of the prognostic capability of the IRM index for OS (3 years, AUC = 0.717; 5 years, AUC = 0.730; 7 years, AUC = 0.731; Figure 3C).

### 3.3 Validation of the predictive ability of the IRM in the meta‐GEO and Nanjing cohorts

To identify the robustness of the IRM index, its capability was evaluated in an independent validation cohort (the meta‐GEO cohort), which included 131 patients with LUAD. Using the same formula and cut‐point as in the TCGA cohort, patients in the meta‐GEO cohort were separated into high‐ and low‐index groups. The results revealed that high‐index patients had outstandingly worse OS compared to patients with a low index, which was consistent with the results of the TCGA cohort (Figure 3D). The IRM index distribution, OS and 6‐gene expression heatmap are shown in Figure 3E. The time‐dependent ROC curve analysis of the IRM index in the meta‐GEO cohort revealed the robustness of the prognostic capability of the IRM index for OS (3 years, AUC = 0.643; 5 years, AUC = 0.635; 7 years, AUC = 0.620; Figure 3F). To better validate the clinical value of the IRM index, we assessed its prognostic capability in the Nanjing cohort, which included 92 patients who were diagnosed with LUAD at the Jiangsu Cancer Hospital and underwent radical surgery without neoadjuvant chemotherapy. The patients in the Nanjing cohort were divided into high‐ and low‐index groups. The Kaplan‐Meier analysis revealed consistent results in the Nanjing cohort (Figure 3G). The IRM index distribution, OS, and 6‐gene expression heatmap are shown in Figure 3H. The time‐dependent ROC curve analysis of the Nanjing cohort revealed the robustness of the prognostic capability of the IRM index for OS (3 years, AUC = 0.705; 5 years, AUC = 0.608; 7 years, AUC = 0.609; Figure 3I).

### 3.4 Validation of the IRM in different clinical subgroups

**FIGURE 4:**
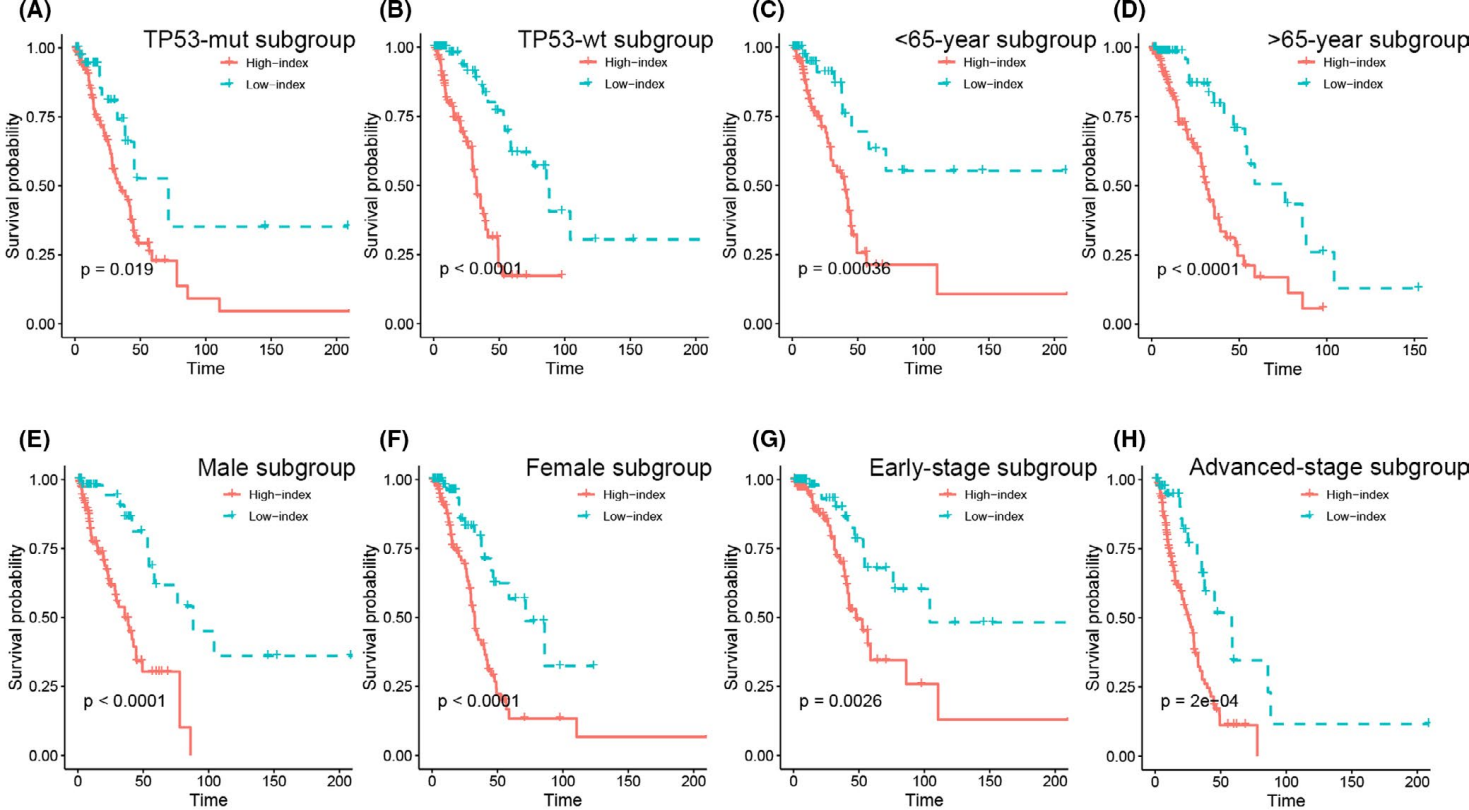
Stratification analysis. The Kaplan‐Meier analysis of the IRM grouping according to patients with (A) TP‐53 mutant, (B) TP‐53 wildtype, (C) <65 years, (D) >65 years, (E) male, (F) female, (G) early stage (TNM stage I), (H) advanced stage (TNM stage II, III, IV)

*TP53* status has a remarkable relationship with the clinical outcome of patients with LUAD. Stratification analyses were used to test the prediction ability of the IRM index in the TP53^mut^ and TP53^wt^ subgroups. As shown in Figure 4A, B, both in the TP53^mut^ and TP53^wt^ subgroups, patients with a high index had worse OS than patients with a low index. Apart from *TP53* status, factors such as age, sex, and tumour‐node‐metastasis (TNM) stage may also affect LUAD prognosis. We illustrated that the IRM index was a robust biomarker for estimating OS in younger (≤65 years) or older (>65 years), male or female, and early tumour stage (TNM stage I) or advanced tumour stage (TNM stage II, III and IV) patients (Figure 4C–H).

### 3.5 Immune status between the low‐ and high‐index patients with LUAD

**FIGURE 5:**
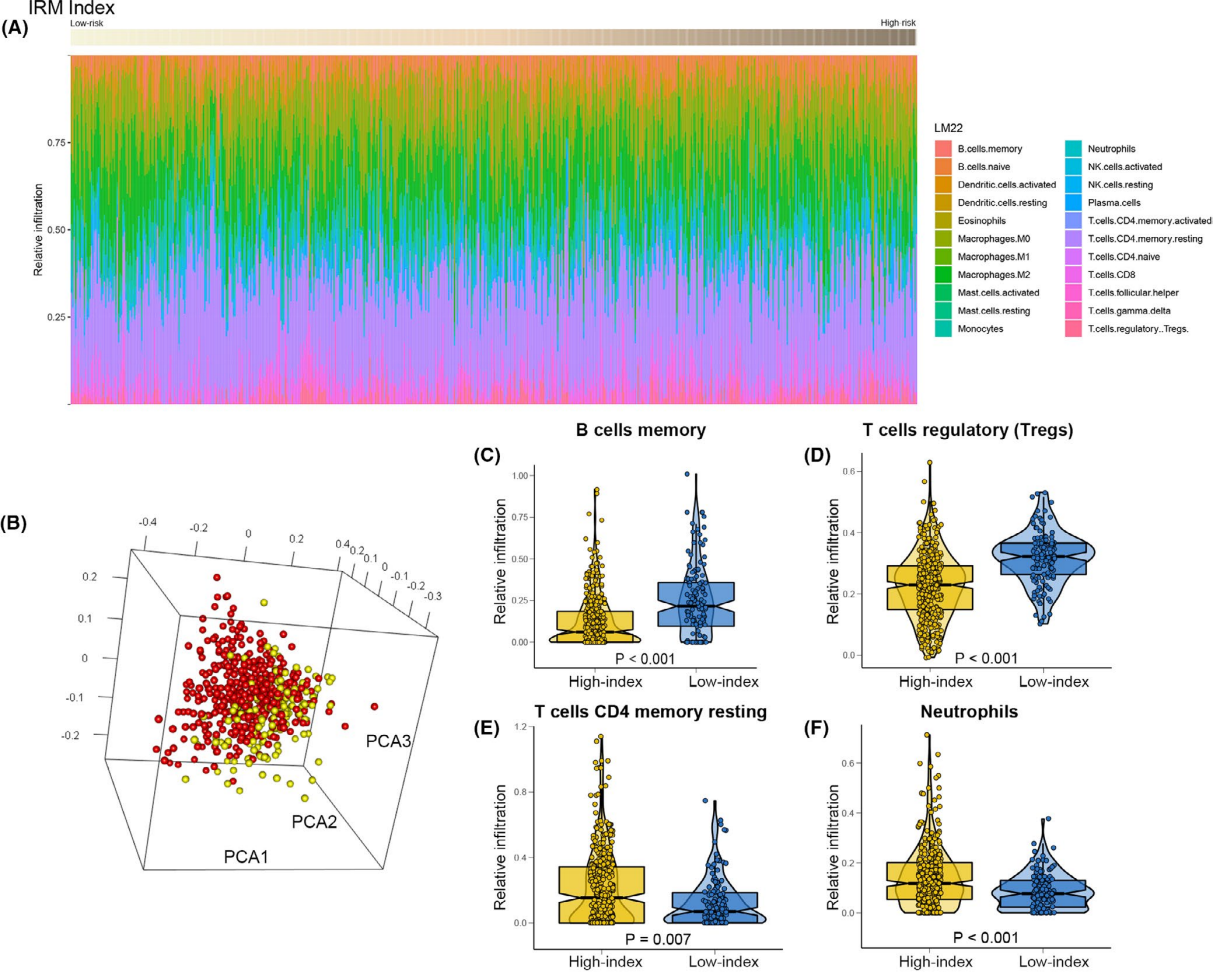
The landscape of immune infiltration in high‐ and low‐index LUAD patients. (A)Relative infiltration of all 22 immune cells in high‐ and low‐index patients. (B) Principal components analysis performed on LUAD patients based on significant differences in immune cells between high‐index and low‐index LUAD patients. Box‐Violin plots visualizing significantly different immune cells: (C) Memory B cells, (D) Regulatory T cells (Tregs), (E) Resting memory CD4+ T cells, (F) Neutrophils cells. The test for association between paired samples used Pearson's correlation coefficient. Two‐tailed statistical *p* values were calculated by a two‐sample Mann‐Whitney test or Student's *t*‐test when appropriate

Characterization of the immune infiltration landscape is important to further explain the potential correlation between the IRM index and tumour humoral immunology by the status of the immune microenvironment. First, using Metascape, we annotated the functions of the 75 IRDEGs, which overlapped with the DEGs and Immport database. These IRDEGs were mainly enriched in terms of antimicrobial humoral response, hormone level regulation, hormone secretion and neuroactive ligand binding (Figure S2A, Figure S2B and Figure S2C). These data thus provided solid evidence of the potential relationship between the IRM index and tumour humoral immunology. Meanwhile, the results prompted us to further explore the correlation between the IRM and immune‐related biological processes. Using CIBERSORTx, we identified the relationships between the IRM index and 22 infiltrating immune cells, which were acquired in the TCGA cohort (Figure 5A). High‐ and low‐index patients were segmented into 2 distinct clusters using PCA based on the relative infiltration of the above‐mentioned cell subpopulations (Figure 5B). Additionally, the relative infiltration of the 22 immune‐infiltrating cells showed mild‐to‐moderate association (Figure S3). The high‐risk patients with LUAD had remarkably lower relative infiltration proportions of memory B cells and regulatory T cells (Tregs; Figure 5C, D) and significantly higher relative infiltration proportions of neutrophils and resting memory CD4+ T cells (Figure 5E, F) than low‐risk patients with LUAD. Thus, the above results suggested that the heterogeneity of tumour immune‐related cell infiltration in LUAD may be a potential prognostic biomarker and target for response after immunotherapy and may have remarkable clinical significance.

**FIGURE 6:**
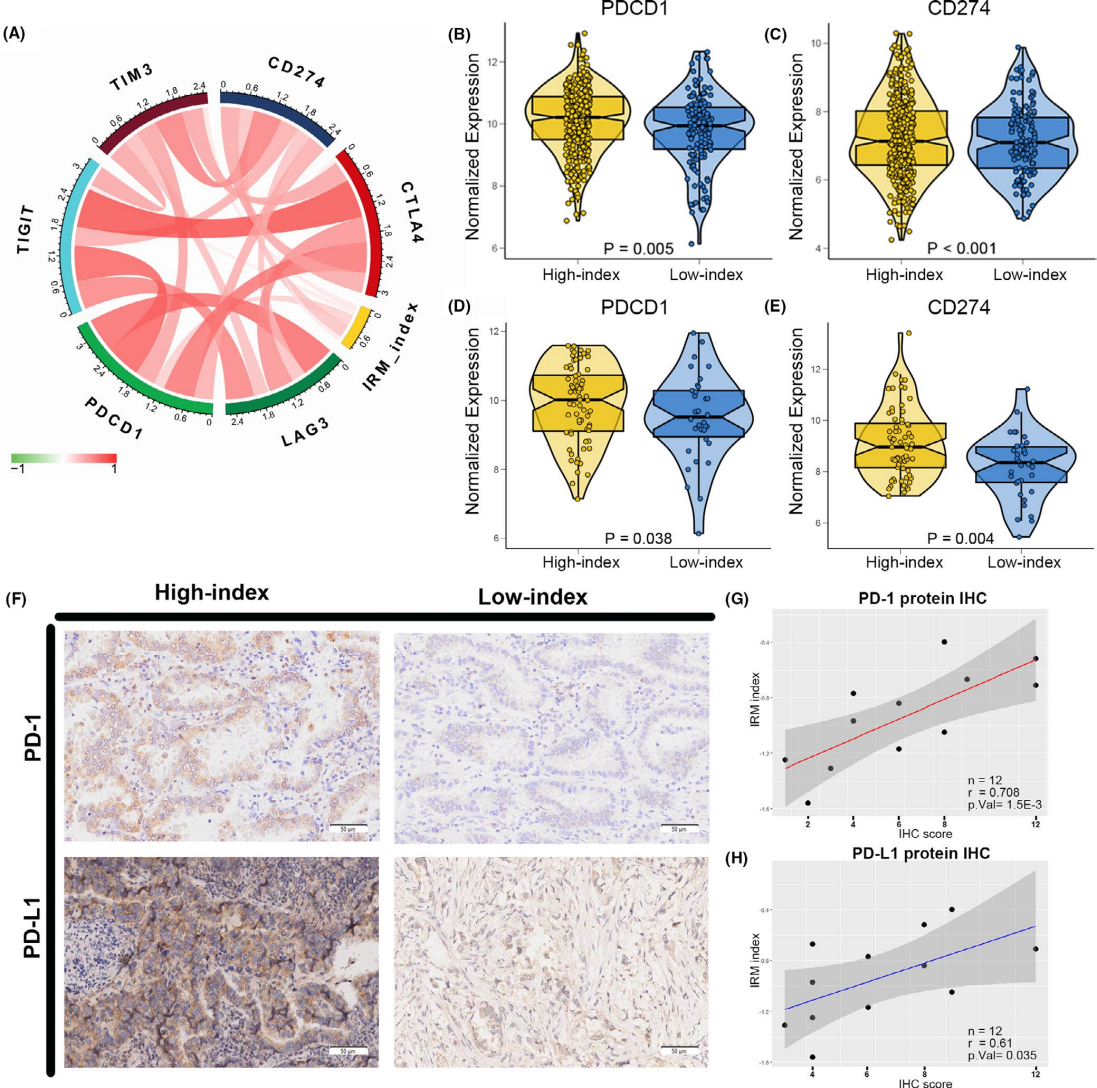
The different expression of immune‐checkpoints between high‐ and low‐ index LUAD patients. (A) Correlation of the IRM index with the expression of several prominent immune‐related checkpoints in TCGA cohort patients. Box‐Violin plots visualizing significantly differently expressed immune‐related checkpoints: (B) PDCD1 expressed and (C) CD274 expressed. Expression of (D) PDCD1 and (E) CD274 between highand low‐index patients in the Nanjing cohort. (F) Representative images of IHC staining of PD‐1 and PD‐L1 in 18 LUAD samples from the Nanjing cohort. (G) (H) The correlation between IRM index and IHC score of the PD‐1, PD‐L1 protein expression

Furthermore, immune checkpoint inhibitors play an antitumour role, reversing the immunosuppressive effect of the tumour. We investigated the correlation between the IRM index and the expression of crucial immune checkpoints, including *PDCD1* (encoding PD‐1), *LAG3*, *TIGIT*, *TIM3*, *CD274* (encoding PD‐L1) and *CTLA4*. The correlation coefficient is shown in Table S5. We observed that the IRM index had a remarkably positive relationship to the expression of *TIM3*, *TIGIT*, *PDCD1* and *CD274* (*p* < 0.05; Figure 6A). Additionally, the relative expression of *PDCD1* and *CD274* in the high‐index group was remarkably higher than in the low‐index group (Figure 6B, C). Moreover, the high‐index patients expressed higher levels of *PDCD1* and *CD274* (Figure 6D, E) in the Nanjing cohort. These results are consistent with a previous study showing that PD‐L1 expression is related to advanced pathological features and worse OS in patients with NSCLC.^15^ We also found the expression differences of PD‐1 and PD‐L1, which are encoded by *PDCD1* and *CD274*, respectively, using IHC samples from 12 patients in the same cohort. These results revealed a significantly positive correlation between the IRM index and IHC scores of these 2 immune checkpoint proteins in patients with LUAD (Figure 6F–H).

### 3.6 Development and verification of a nomogram based on the IRM index

**FIGURE 7:**
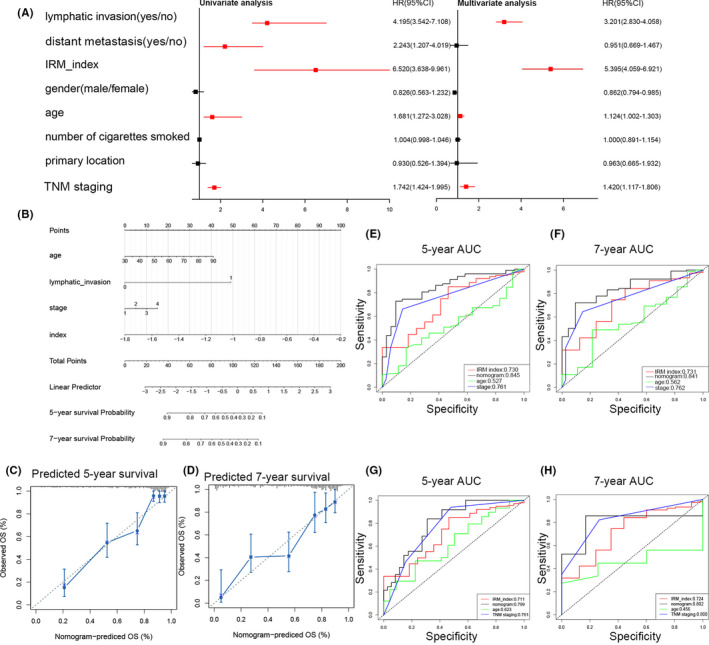
Construction and validation of the nomogram model. (A) Univariate cox regression analyses and (B) multivariate cox regression analyses for lung adenocarcinoma patients in the TCGA cohort. Red indicates statistical significance (*p*‐value < 0.05), and black indicates no statistical significance. (B) Nomogram for predicting the probability of 5‐ and 7‐year OS for lung adenocarcinoma patients of TCGA cohort. The calibration curve of the nomogram for predicting OS at 5‐ (C) and 7‐year (D). Time‐dependent ROC curve analyses of 4 factors, including age, TNM stage, IRM index, and nomogram, in 5 years (E) and 7 years (F) in the TCGA cohort. Validation of time‐dependent ROC curve analyses of 4 factors in 5 years (G) and 7 years (G) in the Nanjing cohort

To assess the prognostic value of the IRM index, a Cox regression analysis was conducted in the TCGA cohort. We first selected the IRM index and several clinical factors for univariate Cox analysis. As shown in Figure 7A, factors including lymphatic invasion, distant metastasis, IRM index, age and TNM stage were significantly related to OS in the TCGA cohort (*p* < 0.05). After multivariate Cox regression analysis of these factors, lymphatic invasion, IRM index, age and TNM stage were still significantly associated with survival (*p* < 0.05). Based on these results, we further integrated the IRM and multiple independent clinical factors (lymphatic invasion, age and TNM stage) to develop a nomogram, which gives clinicians a quantitative way to predict the clinical outcome of patients with LUAD (Figure 7B). The C‐index for the nomogram in the TCGA cohort was 0.746 (95% CI: 0.5886–0.8730). Calibration plots comparing the predicted and actual outcomes of 5‐ and 7‐year OS indicated good agreement (Figure 7C, D). Additionally, we used a time‐dependent ROC curve analysis to compare the robustness of the prognostic capability between the nomogram, IRM index, age, and TNM stage in 1‐, 3‐, 5‐, and 7‐year OS (Figure S4Aand Figure S4B; Figure 7E, F). The nomogram revealed higher prognostic capability with a larger AUC. Furthermore, we verified the prognostic accuracy in the Nanjing cohort. As we expected, the nomogram obtained consistent results (Figure S4C and Figure S4D; Figure 7G, H).

## 4 DISCUSSION

In this study, we systematically analysed the association between *TP53* status and immune‐related phenotypes in patients with LUAD. A *TP53*‐related IRM index was constructed. It was derived from the TCGA cohort and validated in the meta‐GEO and Nanjing cohorts. It was found to be remarkably associated with prognosis. The prognostic value of the 6‐gene IRM index was independent of multiple known strong prognostic factors. Furthermore, the IRM index allowed us to divide patients with LUAD into 2 subgroups with different immune‐related phenotypes. Consequently, we integrated the IRM with multiple clinical factors into a nomogram with robust OS prediction.

Through multi‐functional enrichment analysis of DEGs, we observed obvious enrichment in the humoral immune response. The consistent pathway enrichment analysis results were further verified in selected IRDEGs. Furthermore, 6 IRM‐related genes were found to participate in multiple immunosuppression‐related pathways. Afterwards, we divided the patients with LUAD into 2 subgroups with different prognosis and immune‐related phenotypes. In all 3 cohorts, high‐index patients showed worse survival than low‐index patients. Meanwhile, the IRM index was positively correlated with the expression of several important immune checkpoints. In previous research, NSCLC progression was positively associated with the increased expression of T‐cell exhaustion markers, such as PD‐1, TIM3 and CTLA4,^16^ which is consistent with the results of our research.

CRHR2 belongs to the G‐protein coupled receptor superfamily, regulating corticotropin‐releasing hormone to perform biological functions,^17^ which widely expressed in the gastrointestinal tract, lung and skeletal muscle.^18^ CRHR2 stimulates intracellular cAMP pathway, including activation of nuclear factor‐kB and expression of TNF‐β in T cells.^19^, ^20^ BPIFB1 and BPIFB2 belong to the bactericidal/permeability‐increasing‐fold‐containing family.^21^ BPIFB1 and BPIFB2 protein are most highly expressed in the trachea and lung^22^ and bind to the Gram‐negative bacteria and exert antibacterial function.^23^, ^24^ Moreover, it has been reported that BPIFB1 is abnormally expressed in tumours, which suggests that it plays a role in tumour development.^25^ INHA encodes a member of the TGF‐β superfamily of proteins (RefSeq, Aug 2016), which perform the functions of activated cytokine and hormone.^26^, ^27^ SSTR5 is a predominant component of somatostatin receptor subtypes, which regulates inhibitory effects on endocrine and exocrine secretions.^28^ SSTR5 has a significant regulating‐inflammatory effect via regulating somatostatin, which demonstrated in different animal models.^29^, ^30^ SCGB3A1 belong to secretoglobin gene superfamily, which are cytokine‐like small molecular weight secreted proteins and predominantly expressed in lung airway epithelial cells.^31^ Secretoglobins are thought to be involved in immunomodulatory.^32^ Moreover, SCGB3A1 has been reported as a tumour suppressor in various human tumours including breast, prostate, lung and pancreatic carcinomas.^33^, ^34^

In chronic diseases, T cells malfunction due to T‐cell exhaustion, which increases the expression of inhibitory receptors incorporating PD‐1, LAG3, TIM3, CTLA4 and TIGIT,^35^, ^36^, ^37^, ^38^, ^39^ resulting in fewer cytokines and loss of antitumour capabilities. The limited efficacy of immunotherapy may be due to the production of dysfunctional T cells in the TME.^40^ Therefore, regulators that reverse the state of T‐cell dysfunction are the focus of current research. For example, tertiary lymphoid structure immune activity dysfunction is reversed and antitumour capabilities are enhanced after treatment with anti‐PD‐1/PD‐L1 and anti‐CTLA‐4 immune checkpoint inhibitors in humans and mice.^41^, ^42^, ^43^, ^44^ The PD‐L1/PD‐1 axis is an important regulatory pathway of T‐cell exhaustion in tumours. DD1α expression induced by p53, which is encoded by *TP53*, has been shown to upregulate PD‐1 and PD‐L1, as cancer cells respond to genotoxic stress and DNA damage, which then promotes the gradual priming of immune surveillance.^45^ This finding clarified the relationship between p53 and immune checkpoint inhibitors, which may indicate the intrinsic molecular mechanism of the relationship between the IRM index and the expression of immune checkpoints. PD‐L1 has abundant expression in cancer cells and the tumour extracellular matrix, and blocking the PD‐L1/PD‐1 axis can enhance the antitumour capabilities of T cells.^46^ In our study, the IRM index was positively correlated with the expression of immune checkpoints, which shows that as the IRM index grows, T cells are exhausted and their antitumour abilities decrease. This explains the poor prognosis of high‐index patients with LUAD. It can be speculated that immunotherapies that block the pathways that suppress tumour immune responses for patients with LUAD in the high‐index group may increase the presentation of cancer‐associated antigens, resulting in the recovery of the immune response of CD8+ T cells,^47^ which may then result in better immunotherapy effects.

In the immune infiltration analysis between the high‐ and low‐index patients, the high‐index patients with LUAD had remarkably higher proportions of neutrophils and resting memory CD4+ T cells and lower proportions of memory B cells and Tregs. Memory resting CD4+ T cells can be further differentiated into multiple cell subpopulations and confer different functions, including blocking CD8+ T‐cell activation and NK cell killing and suppressing the immune response to autoantigens and exogenous antigens.^48^ In human NSCLC, neutrophils play a key role in tumour immunity.^49^ In mice, neutrophils infiltrating tumours can either promote carcinogenesis by supporting tumour‐related inflammation, angiogenesis and metastasis and inhibiting T‐cell activation or restrict tumour growth through the expression of antitumour and cytotoxic mediators.^50^ Nevertheless, because the tumour is in an irreversible continuous chronic inflammatory state, suppressive neutrophils are constantly mobilized and become the dominant subpopulation of neutrophils,^51^ which may suppress the immune response and promote malignant progression. In our study, the high‐index patients with LUAD had significantly higher proportions of neutrophils and resting memory CD4+ T cells, restricting the function of CD8+ T cells and NK cells in the tumour development process, resulting in malignant tumour progression and worse OS. It is reasonable to speculate that the high‐index patients with LUAD may have improved CD8+ T‐cell function and receive better efficacy by using an immune checkpoint inhibitor that blocks the PD‐1/PD‐L1 axis.

Tregs are an inhibitory subpopulation of CD4+ T cells. Cancer‐associated fibroblasts in the extracellular matrix express COX‐2, which promotes PGE2 secretion to induce immunosuppressive FOXP3+ Tregs,^52^ which then accumulate in primary tumour tissues and the peripheral blood to promote immune evasion.^46^ Progressing tumours can inhibit CD8+ T cells through several approaches, including Tregs, which can directly suppress the antitumour functions of CD8+ T cells.^53^ The immunosuppressive state of the high‐index patients with LUAD may not through the suppression of CD8+ T cells immune response by Tregs. Tumour‐infiltrating B cells are a key component of the TME. B cells and CD4+ T cells together form tertiary lymphoid structures, which are related to better outcomes.^54^ This is consistent with the results of our study, as low‐index patients had better prognoses. A variety of cytokines, including tumour necrosis factor (TNF), interleukin (IL)‐2, IL‐6 and interferon gamma (IFNγ), which are secreted by B cells, could assist B cells to recruit other immune effector cells and activate immune responses, including T cells. Memory B cells may play a role in inhibiting antigen presentation, thereby driving the expansion of memory and naive T‐cell responses. Memory B cells and plasma cells show similar characteristics of facilitating an acquired immune response, which also contributes to an effective T‐cell response after immune checkpoint inhibitor therapy.^55^ The high‐index patients with LUAD had lower proportions of memory B cells, indicating that the suppression of T‐cell antitumour functions in such patients may be due to the decline of memory B cell antigen presentation functions, which is related to worse OS.

Our research supplies a novel angle regarding LUAD immune microenvironment groupings and immunotherapy responses. However, since our study is a retrospective study, it has limitations and needs to be further validated by prospective studies. Furthermore, mechanistic studies of the IRM index‐related genes and immune‐infiltrating cells need to be implemented to explain their clinical application. In subsequent research, we will focus on single‐cell transcriptome studies of immune‐infiltrating cells in patients with LUAD with different IRM indexes.

In conclusion, the IRM index is a robust clinical biomarker that can assign patients with LUAD to subgroups with significantly different prognoses and immune‐related phenotypes, which may explain the molecular mechanism of different prognoses from the perspective of immunology.

## CONFLICT OF INTEREST

The authors declare that the research was conducted in the absence of any commercial or financial relationships that could role as a potential conflict of interest.

## AUTHOR CONTRIBUTIONS

**Xuming Song:** Data curation (lead); Investigation (equal); Methodology (lead); Writing – original draft (equal). **Qiang Chen:** Data curation (equal); Project administration (equal); Writing – original draft (equal). **Jifan Wang:** Investigation (lead); Methodology (equal); Validation (lead). **Qixing Mao:** Software (lead); Supervision (supporting); Validation (equal). **Wenjie Xia:** Supervision (equal). **Lin Xu:** Funding acquisition (supporting); Supervision (supporting). **Feng Jiang:** Funding acquisition (lead); Supervision (lead); Writing – review & editing (lead). **Gaochao Dong:** Funding acquisition (equal); Supervision (equal); Writing – review & editing (equal).

## Supporting information

Figure S1

Figure S2

Figure S3

Figure S4

Table S1

Table S2

Table S3

Table S4

Table S5

## Data Availability

The data sets supporting the conclusions of this article are available from the ‘TCGAbiolinks’ R package (version 2.14.1; https://bioconductor.org/packages/release/bioc/html/TCGAbiolinks.html ) and GEO database (https://www.ncbi.nlm.nih.gov/gds).
