# Supplementary figures and images for: Clinical and prognostic implications of an immune‐related risk model based on TP53 status in lung adenocarcinoma
Source: J Cell Mol Med. 2021 Dec 8;26(2):436–48. doi: 10.1111/jcmm.17097 (PMC8743672; doi:10.1111/jcmm.17097)

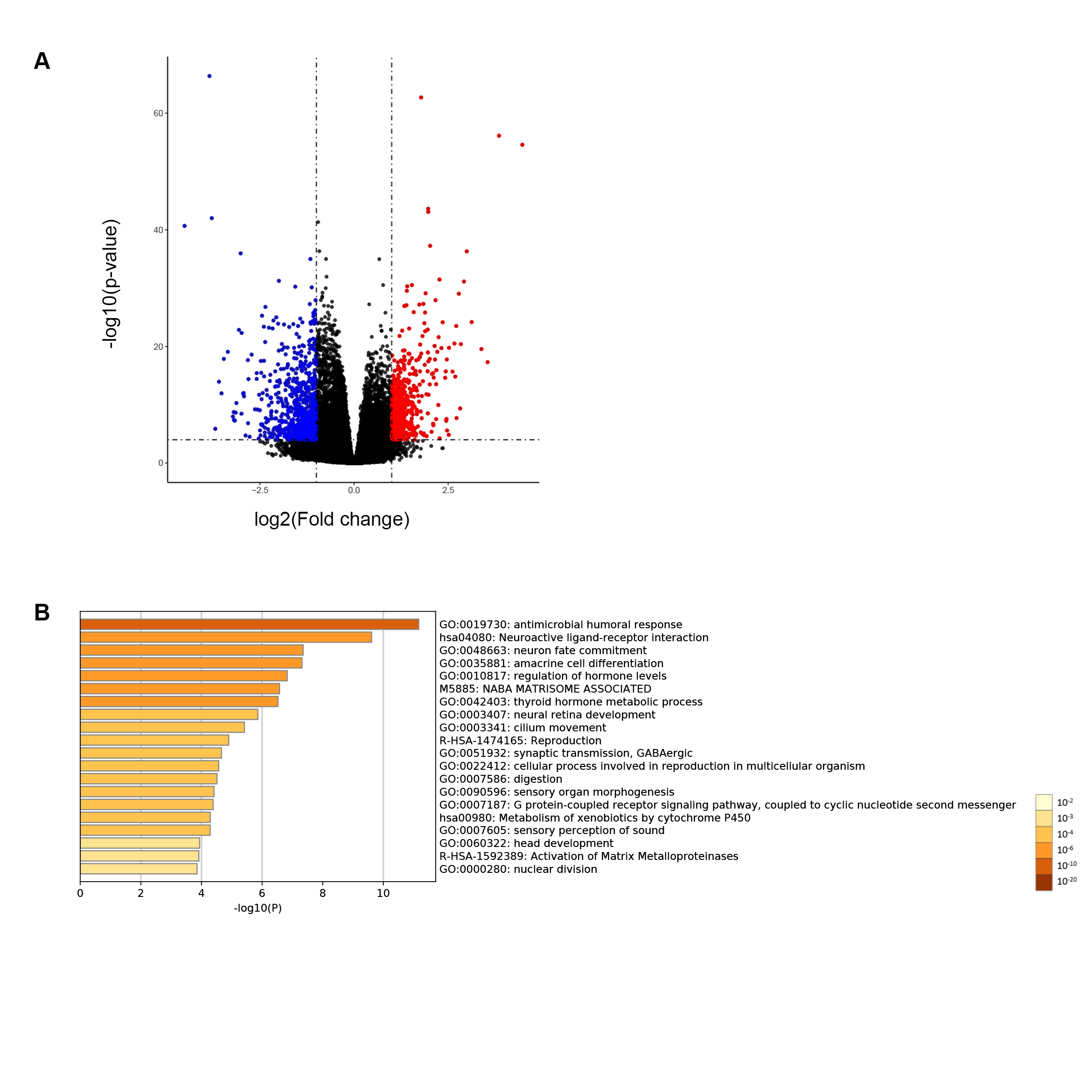

Supplement: Supplementary file 1 — Figure S1 [file JCMM-26-436-s008.tif]

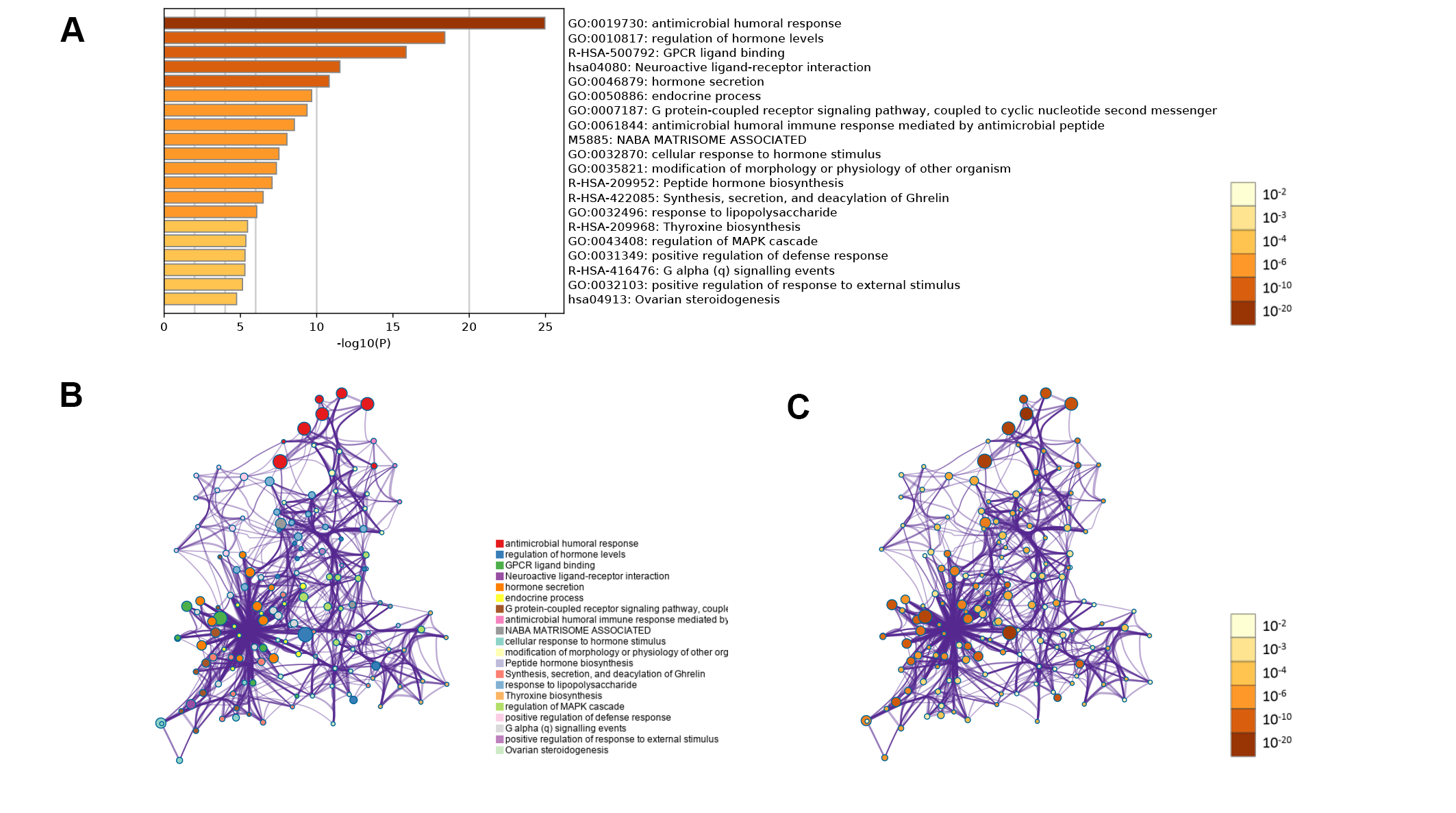

Supplement: Supplementary file 2 — Figure S2 [file JCMM-26-436-s009.tif]

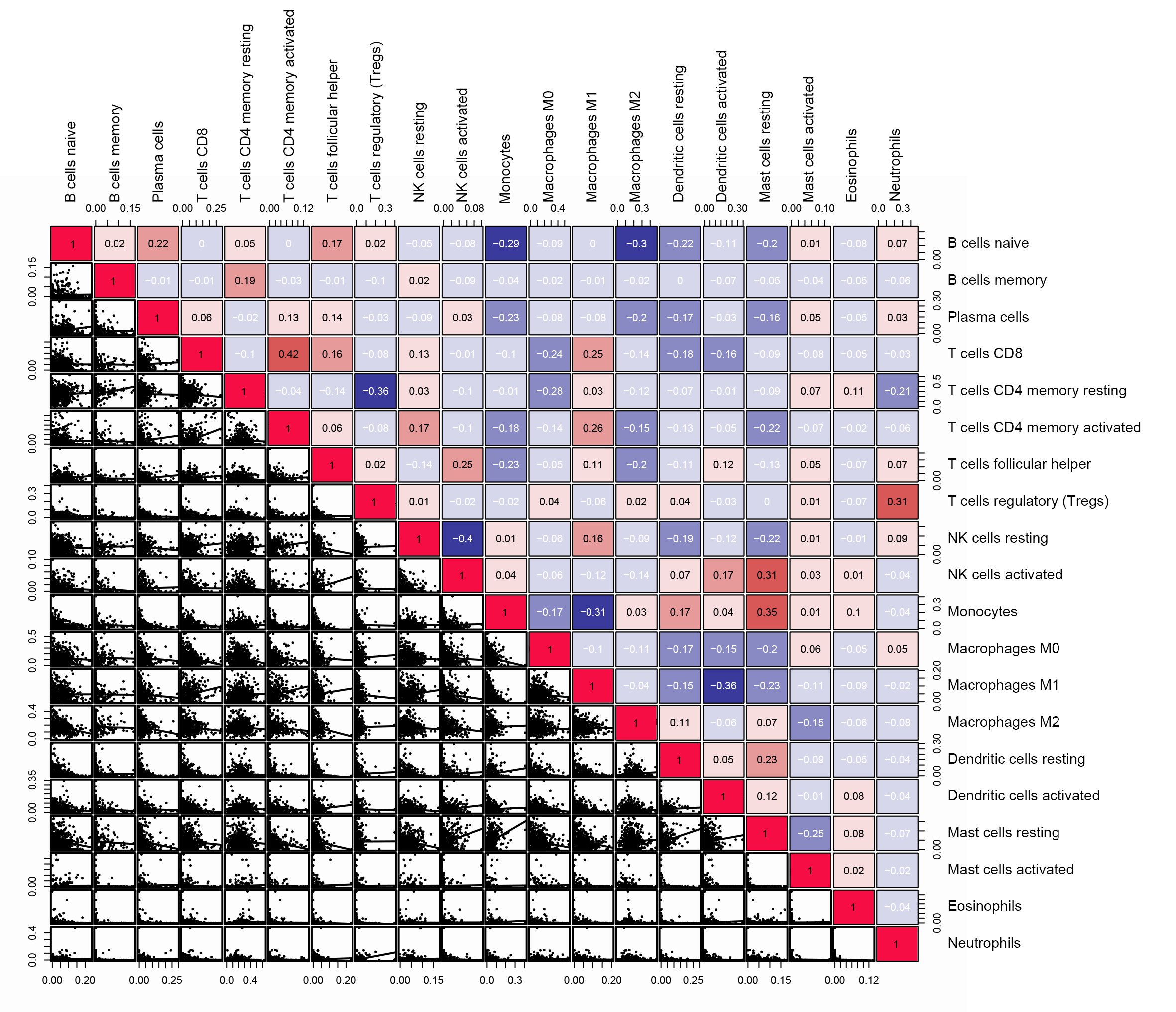

Supplement: Supplementary file 3 — Figure S3 [file JCMM-26-436-s005.tif]

**A** 1-year ROC (TCGA)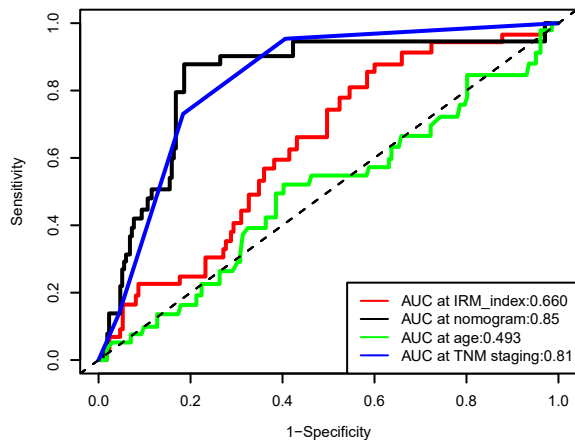**B** 3-year ROC (TCGA)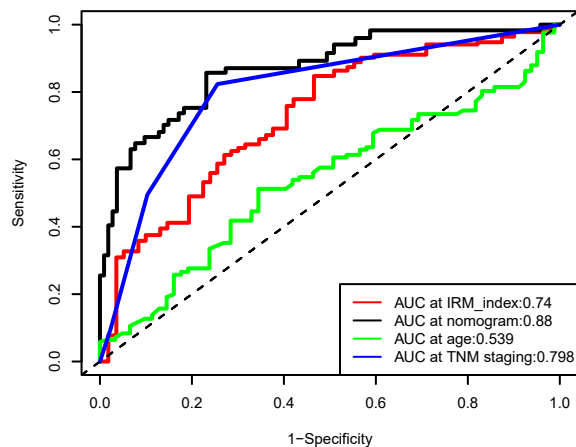**C** 1-year ROC (Nanjing)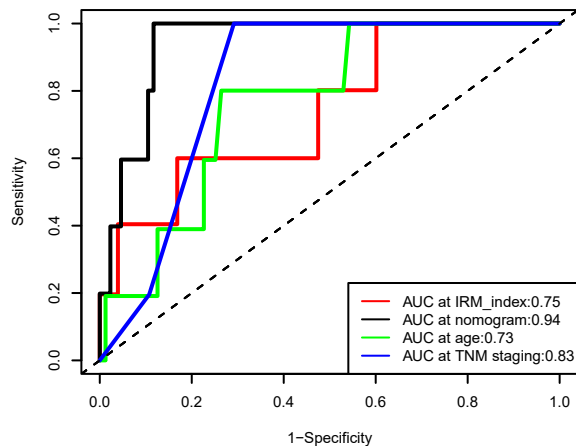**D** 3-year ROC (Nanjing)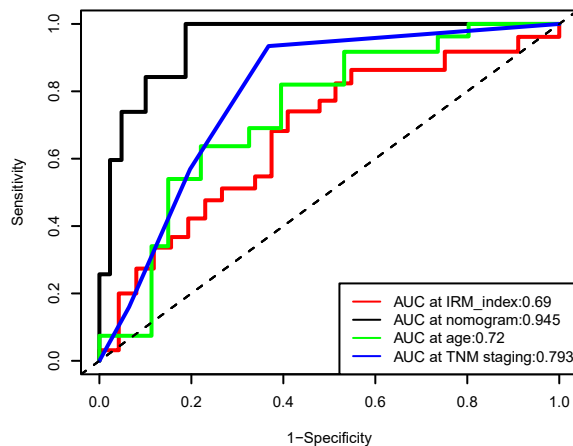

Supplement: Supplementary file 4 — Figure S4 [file JCMM-26-436-s001.pdf]
