# Supplementary material for: Clinical and prognostic implications of an immune‐related risk model based on TP53 status in lung adenocarcinoma
Source: J Cell Mol Med. 2021 Dec 8;26(2):436–48. doi: 10.1111/jcmm.17097 (PMC8743672; doi:10.1111/jcmm.17097)
Supplement: Supplementary file 5 — Table S1 [file JCMM-26-436-s006.docx]

**Supplementary Table 1** Clinical character of LUAD patients in the Nanjing cohort

| **Clinical character** |  | **Total (percentage)** |
| --- | --- | --- |
| **Age** | ≤65  >65 | n=49 (53.3%)  n=43 (46.7%) |
| **Gender** | Female  Male | n=50 (54.3%)  n=42 (45.7%) |
| **Primary tumor (T)** | T1  T2  T3  T4 | n=42 (45.7%)  n=20 (21.7%)  n=20 (21.7%)  n=10 (10.9%) |
| **Regional Lymph node invasion (N)** | Yes  No | n=42 (45.7%)  n=50 (54.3%) |
| **Distant metastasis (M)** | Yes  No | n= 9 (9.7%)  n=83 (90.3%) |
| **Living status** | Alive  Dead | n=57 (62.0%)  n=35 (38.0%) |
